# 919. Household Flu Transmission and Healthcare Resource Use among Patients Treated with Baloxavir versus Oseltamivir for Influenza: An Outpatient Prospective Survey in the United States

**DOI:** 10.1093/ofid/ofad500.964

**Published:** 2023-11-27

**Authors:** Jennie H Best, Mitra H Sadeghi, Xiaowu Sun, Arpamas H Seetasith, Lisa Albensi, Seema Joshi, Marcus Zervos

**Affiliations:** Genentech, Inc., South San Francisco, California; Genentech, Inc., South San Francisco, California; CVS Health Clinical Trial Services, New York, New York; Genentech, Inc., South San Francisco, California; CVS Health Clinical Trial Services, New York, New York; Henry Ford Hospital, Detroit, Michigan; Henry Ford Hospital, Detroit, Michigan

## Abstract

**Background:**

Influenza is a common seasonal infectious disease that has broad medical, economic, and social consequences. Understanding how flu antiviral use affects transmission to household (HH) members is critical to inform clinical practice. Objectives of this study were to compare the incidence of HH transmission (primary) and healthcare resource utilization (HRU, secondary) in influenza patients treated with baloxavir (BALOX) compared with oseltamivir (OSELT). This is the first analysis to examine the effectiveness of BALOX vs OSELT for reducing transmission in a real-world setting in the US.

**Methods:**

Patients were recruited from October 2022 to March 2023 via CVS Pharmacy in the US. Patients were eligible for inclusion if: ≥ 18 years, filled an Rx for BALOX or OSELT ("index" day) ≤ 2 days from flu symptom onset and also reported taking the therapy, had no HH flu ≤ 7 days pre-index, had no COVID ≤ 30 days pre-index, and had ≥ 2 persons in HH. Patients were contacted via email or call center from Days 4 though 8 post-index to inform and/or remind patients about the online survey. Patients completed the survey on Days 6, 7, or 8 post-index. BALOX-treated patients were matched (non-randomized) with up to 3 OSELT-treated patients on same RX fill date. Household transmission (reported by index patient) and all-cause HRU (of the index patient) in the 8 days post-index were assessed using chi-square and Fisher's exact tests.

**Results:**

Of 77,314 unique patients contacted, 1,301 (1.7%) patients consented. Of 356 eligible patients, 275 (89 BALOX-treated and 186 OSELT-treated patients) completed the survey and were included in the analysis. Patients were mean age 45, female (65%), and White (87%). Lower HH transmission was observed with BALOX compared with OSELT therapy (18.0% vs 27.4%; standardized mean difference = 0.23; RR = 0.66; p= 0.09; Figure 1). HRU was also numerically lower in the BALOX-treated group, particularly emergency department visits (0.0% vs 4.3%; p = 0.06; Table 1); no hospitalizations were reported.

**Figure:**
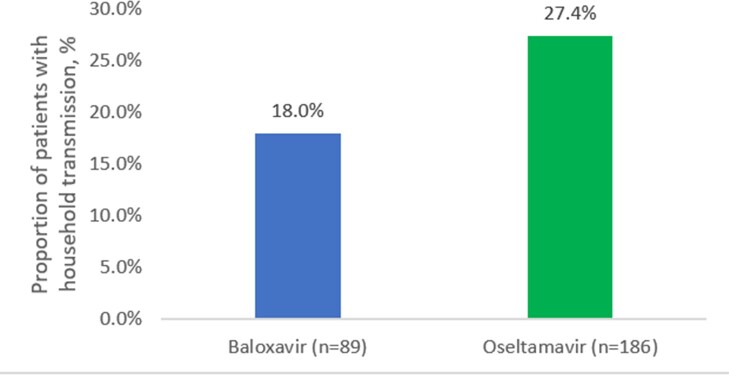

Proportion of patients with healthcare resource utilization

**Figure:**
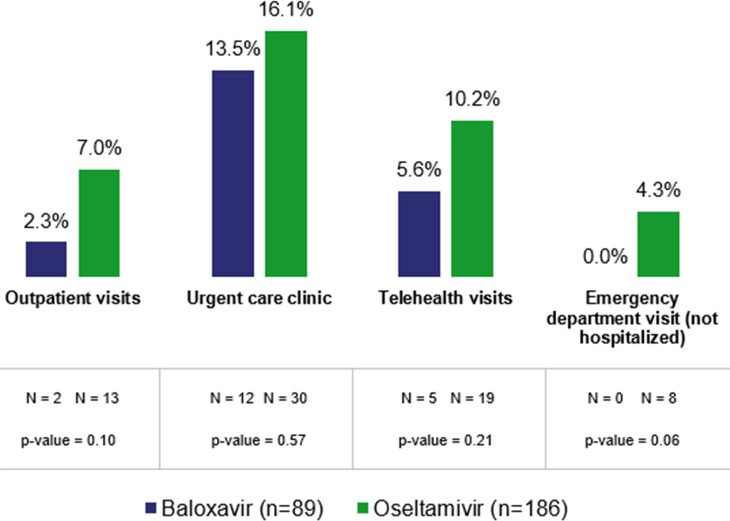

**Conclusion:**

The findings in this real-world study suggest that treatment of influenza with BALOX may decrease transmission to household members and reduce healthcare resource utilization compared with OSELT treatment. Future research could validate these findings in larger populations.

**Disclosures:**

**Jennie H. Best, PhD**, Genentech: Stocks/Bonds **Mitra H. Sadeghi, PharmD**, Genentech, Inc.: Stocks/Bonds **Xiaowu Sun, PhD**, CVS: Stocks/Bonds **Arpamas H. Seetasith, PhD**, Genentech, Inc.: Stocks/Bonds **Marcus Zervos, MD**, Contrafect: Advisor/Consultant|GSK: Grant/Research Support|Johnson and Johnson: Grant/Research Support|Pfizer: Grant/Research Support

